# Associations among Erythroferrone and Biomarkers of Erythropoiesis and Iron Metabolism, and Treatment with Long-Term Erythropoiesis-Stimulating Agents in Patients on Hemodialysis

**DOI:** 10.1371/journal.pone.0151601

**Published:** 2016-03-15

**Authors:** Hirokazu Honda, Yasuna Kobayashi, Shoko Onuma, Keigo Shibagaki, Toshitaka Yuza, Keiichi Hirao, Toshinori Yamamoto, Naohisa Tomosugi, Takanori Shibata

**Affiliations:** 1 Division of Nephrology, Department of Medicine, Showa University Koto Toyusu Hospital, Tokyo, Japan; 2 Division of Clinical Pharmacy, Department of Pharmacotherapeutics, Showa University, School of Pharmacy, Tokyo, Japan; 3 Division of Nephrology, Department of Medicine, Showa University, School of Medicine, Tokyo, Japan; 4 Shibagaki Dialysis Clinic Jiyugaoka, Tokyo, Japan; 5 Shibagaki Dialysis Clinic Togoshi, Tokyo, Japan; 6 Aging Research Unit, Department of Advanced Medicine, Medical Research Institute, Kanazawa Medical University, Kanazawa, Japan; Lady Davis Institute for Medical Research/McGill University, CANADA

## Abstract

**Background:**

We aimed to identify associations between erythroferrone (ERFE), a regulator of hepcidin 25, and biomarkers of erythropoiesis and iron metabolism. We also aimed to determine the effects of erythropoiesis-stimulating agents (ESA), continuous erythropoietin receptor activator (CERA) and darbepoetin-α (DA) on ERFE production in patients on hemodialysis (HD).

**Methods:**

Blood samples were obtained from 59 patients before HD sessions on day 0 (baseline). Twenty patients who were injected with either CERA (N = 10) or DA (N = 10) at the end of the dialysis week (day 0), who had ferritin ≥ 100 ng/mL and/or transferrin saturation ≥ 20%, and hemoglobin > 9 g/dL were selected from among the 59 patients. Blood was sampled serially before HD sessions on days 3, 5, 7 from patients on DA and on the same days plus day 14 from those on CERA.

**Results:**

Levels of ERFE correlated inversely with those of hepcidin 25 and ferritin, and positively with those of soluble transferrin receptor. The hepcidin 25: ERFE ratio and hepcidin 25 levels positively correlated with ferritin levels. Levels of ERFE significantly increased from day 3 of treatment with DA and CERA and decreased by days 7 and 14, respectively. Erythropoiesis-stimulating agents concomitantly decreased levels of hepcidin 25 as those of ERFE increased.

**Conclusion:**

We identified a novel association between ESA and ERFE in patients on HD. Both DA and CERA increased levels of ERFE that regulated hepcidin 25 and led to iron mobilization from body stores during erythropoiesis.

## Background

Anemia in patients with chronic kidney disease (CKD) is characterized by reduced renal erythropoietin (EPO) production and decreased red-cell survival [1]. Moreover, patients undergoing dialysis are often iron-deficient [2]. Adequate iron stores must be maintained to ensure effective treatment of renal anemia using erythropoiesis-stimulating agents (ESA) because decreased iron stores or availability comprise the most common reasons for resistance to ESA [2].

Several studies have confirmed a relationship between ESA and biomarkers of iron metabolism in patients under dialysis, and ESA impact biomarkers of iron metabolism including hepcidin 25, the key regulator of stored iron release [3–6]. Erythropoiesis-stimulating agents significantly suppress levels of hepcidin 25 and ferritin, and therapy with long-acting ESA results in effective erythropoiesis and stored iron release [3–6].

During erythropoiesis, erythroblasts produce erythroferrone (ERFE), encoded by the FAM132B gene, which regulates hepcidin 25 production and thus iron metabolism [7, 8]. Thus, ERFE might be a key factor in the control of stored iron release. Levels of ERFE in patients with breast cancer have recently been reported [9]. However, associations between ERFE and biomarkers of erythropoiesis and iron metabolism, and the ability of ESA to increase ERFE levels in terms of its onset and duration of action on iron metabolism have not been evaluated. Therefore, the present study aimed to determine associations between ERFE and biomarkers of erythropoiesis and iron metabolism, and to analyze the effects of the long-acting ESA, darbepoetin-α (DA) and of a continuous erythropoietin receptor activator (CERA) in terms of iron utilization for erythropoiesis via ERFE production in patients on hemodialysis (HD).

## Methods

### Patients

Our Institutional Committee on Human Research approved the protocol of the study, which proceeded according to the 2008 revision of the Declaration of Helsinki. The present prospective observational study included a cross-sectional analysis at baseline. Fifty-nine outpatients (aged ≥ 20 years) who had been receiving maintenance HD and ESA therapy at two clinics (Shibagaki Dialysis Clinic, Jiyugaoka and Shibagaki Dialysis Clinic, Togoshi) for over six months provided written, informed consent to participate in the study. Patients with malignant, chronic inflammatory, or severe liver or lung diseases, and those under treatment with anti-inflammatory or immunosuppressive agents were excluded. Ten patients each who were under treatment with ESA, DA or CERA, who were not iron-deficient (ferritin ≥ 100 ng/mL and/or transferrin saturation ≥ 20%) and hemoglobin > 9 g/dL were selected from among the 59 patients. All patients received standard-of-care treatment according to their clinical presentations as described below.

### Administration of ESA

The enrolled patients had been receiving intravenous CERA, Mircera (Chugai Pharmaceutical Co. Ltd., Tokyo, Japan) once every two or four weeks, or DA (Nesp; Kyowa Hakko Kirin Co. Ltd., Tokyo, Japan) once per week for the past three months. Anemia was managed according to the Guidelines for Renal Anemia published by the Japanese Society for Dialysis Therapy [10]. The ESA dose was controlled to maintain a target hemoglobin range of 10.0–11.0 g/dL. The patients were administered with the same doses of DA and CERA on day 0 (baseline) that they had previously received.

### Iron supplementation

A 40-mg dose of saccharated ferric oxide (Fesin; Nichiiko Pharmaceutical, Toyoma City, Japan) was administered intravenously once weekly during the study if hemoglobin levels decreased to < 10 g/dL and those of serum TSAT and ferritin reached < 20% and < 100 ng/mL, respectively [10].

### Blood sampling

Venous blood was sampled from 59 patients injected with ESA on the last day of the dialysis week (day 0), which was before the HD session on the third day of the week, on days 3, 5 and 7 thereafter from patients on DA and on the same days plus day 14 from patients with CERA. The controls comprised stored venous blood samples collected from 13 individuals with normal kidney function who provided written, informed consent.

### Measurements

Routine biochemical parameters and levels of high-sensitive C-reactive protein (hsCRP), serum iron, total iron-binding capacity (TIBC), ferritin, soluble transferrin receptor (sTfR), ERFE and hepcidin 25 were measured in venous blood samples obtained at each time point. Serum samples were immediately frozen and stored at -80°C before analyses of sTfR using human sTfR immunoassays (R&D Systems, Minneapolis, MN, USA), hepcidin 25 using liquid chromatography-tandem mass spectrometry [11] and ERFE using a human protein FAM132B ELISA kit (MyBioSource, San Diego, CA, USA), with an intra-CV and inter-CV values of 7.6% and 10.4%, respectively.

### Statistical analysis

Data are presented as means ± SD or as medians (range) unless otherwise stated, with *P* < 0.05 considered to indicate statistical significance. Normally distributed variables were compared between the two groups using Student’s *t*-test, and non-normally distributed variables were analyzed using the Wilcoxon rank-sum test. Nominal variables were compared between the two groups using Fisher’s exact test. The strength of a linear association between two variables was measured using Pearson’s correlation coefficient. Trends of changes in parameters caused by injected DA or CERA were assessed using Friedman’s test and parameters were compared between baseline and each time point after DA or CERA injection using the Wilcoxon matched-pairs signed-rank test. Data were analyzed using JMP Pro 11.0 (SAS Institute, Cary, NC, USA) and Prism 5.0c (GraphPad Software Inc., La Jolla, CA, USA).

## Results

Mean levels of ERFE in blood samples from patients under HD at baseline (n = 59) and from controls with normal kidney function (n = 12) did not significantly differ (484.6 ± 153.3 vs. 503.6 ± 60.6 pg/mL).

### Baseline analysis

**Fig 1:**
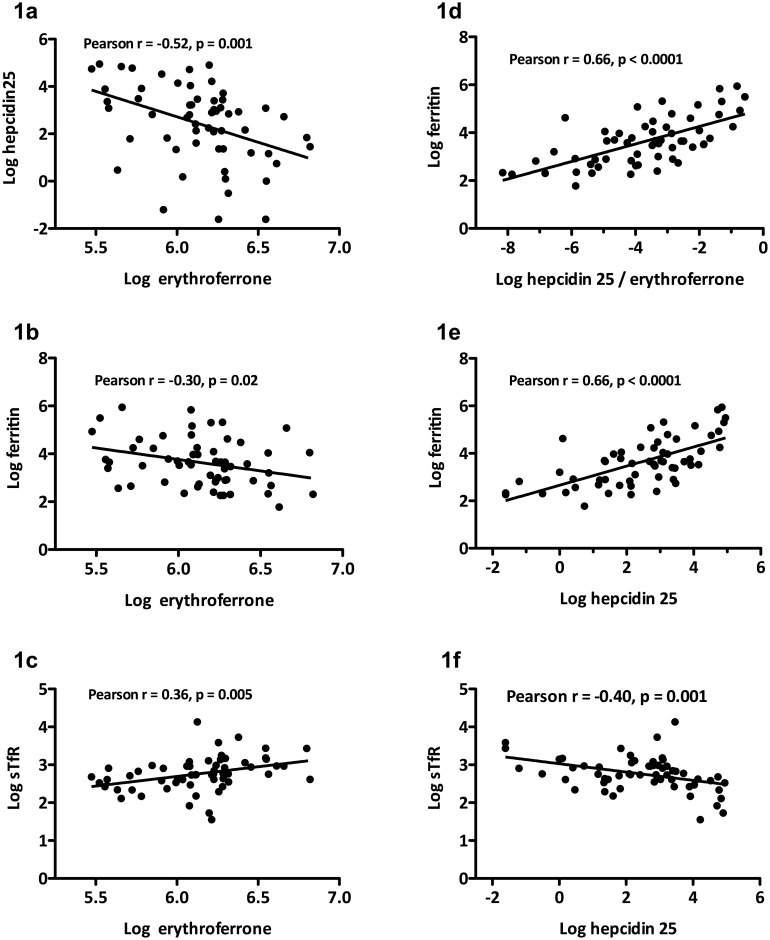
Associations among erythroferrone, hepcidin 25, ratios of hepcidin 25 to erythroferrone, ferritin, and soluble transferrin receptor (sTfR) in patients under hemodialysis. Data are log-transformed.

Table 1 shows the baseline characteristics of the patients. Levels of ERFE correlated inversely with hepcidin 25 and ferritin, and positively with sTfR (Fig 1). Ratios of hepcidin 25 to ERFE and levels of hepcidin 25 correlated positively with those of ferritin (Fig 1). Log-transformed ERFE correlated positively with log-transformed hs-CRP (Pearson r = 0.33, p = 0.01).

**Table 1:** Baseline patient characteristics.

	Total (n = 59)	DA^1^ (n = 10)	CERA^2^ (n = 10)	P^3^
Age (y)	66 ± 11^4^	69 ± 11	71 ± 8	0.85
Gender (male %)	59	80	50	0.10
Body mass index (kg/m^2^)	21.3 ± 3.6	21.2 ± 2.8	21.3 ± 4.7	0.73
Diabetes mellitus (%)	26	30	20	0.60
Hemodialysis vintage (months)	71 (12, 347) ^5^	60 (13, 248)	95 (12, 347)	0.12
Kt/V	1.4 ± 0.2	1.4 ± 0.3	1.4 ± 0.2	0.72
Mean ESA dose (μg)	-	20 ± 8	63 ± 32	-
Patients on ferrotherapy (%)	15	30	20	0.60
Albumin (g/mL)	3.8 ± 0.2	3.8 ± 0.4	3.8 ± 0.3	0.83
Creatinine (mg/dL)	10.5 ± 2.5	10.3 ± 3.0	10.9 ± 2.1	0.32
High-sensitive CRP (mg/dL)	0.19 ± 0.32	0.12 ± 0.14	0.05 ± 0.03	0.19
Hemoglobin (g/dL)	11.0 ± 0.9	10.6 ± 0.6	10.3 ± 0.8	0.39
MCV^6^ (fL)	97.0 ± 6.5	95.9 ± 5.4	96.5 ± 6.1	0.57
MCHC^7^ (%)	31.8 ± 1.1	32.5 ± 1.2	31.8 ± 0.8	0.09
Reticulocyte counts (10^4^/μL)	3.7 ± 1.4	4.1 ± 2.6	2.6 ± 1.7	0.07
Iron (μg/dL)	72.8 ± 26.9	63.5 ± 12.7	77.8 ± 24.5	0.24
Transferrin saturation (%)	26.7 ± 9.1	24.8 ± 4.3	30.6 ± 10.0	0.11
Ferritin (ng/mL)	64.4 ± 77.8	126.0 ± 95.3	120.1 ± 116.2	0.48
sTfR^8^ (mmol/L)	17.6 ± 9.1	17.1 ± 9.7	14.5 ± 8.4	0.44
Hepcidin 25 (ng/mL)	28.9 ± 37.7	42.1 ± 40.1	60.6 ± 58.1	0.78
Erythroferrone (pg/mL)	484.6 ± 153.3	470.3 ± 84.1	509.3 ± 174.0	0.62

^1^Darbepoetin-α;

^2^continuous erythropoietin receptor activator;

^3^DA vs. CERA;

^4^mean ± SD;

^5^median and range;

^6^mean corpuscular volume;

^7^mean corpuscular Hb concentration;

^8^soluble transferrin receptor.

### Erythropoiesis during the study period

Baseline characteristics did not significantly differ between the DA (n = 10) and CERA (n = 10) groups (Table 1). Hemoglobin levels in both groups were controlled within a hemoglobin target of 10.0–11.0 g/dL using DA for seven days and CERA for 14 days (S1a and S2a Figs). Reticulocyte counts were significantly increased on day 5 and persisted until day 7 after DA and CERA administration, respectively (S1b and S2b Figs).

### Changes in biomarkers of iron metabolism in DA and CERA treatment

**Fig 2:**
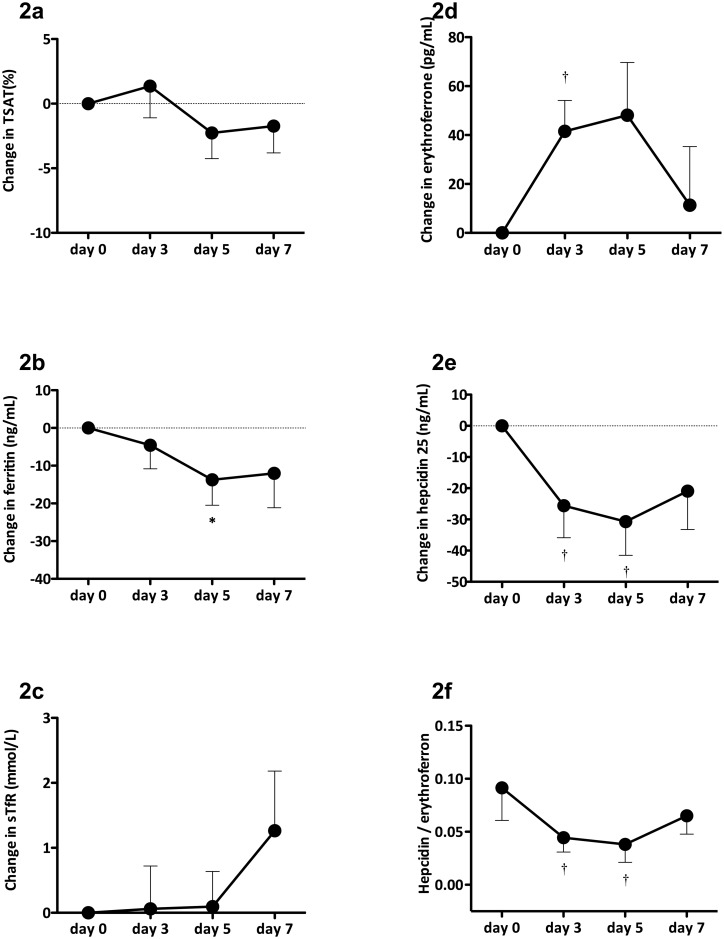
Changes in biomarkers of iron metabolism induced by darbepoetin-α. Biomarkers of iron metabolism; namely transferrin saturation (TSAT) (a), ferritin (b), soluble transferrin receptor (sTfR) (c), erythroferrone (d), hepcidin 25 (e) and ratio of hepcidin 25 to erythroferrone (f). Data are shown as means ± SEM. Baseline and time-point data were compared using Wilcoxon matched-pairs signed-rank test. *p < 0.05 and ^†^p < 0.01 vs. baseline.

**Fig 3:**
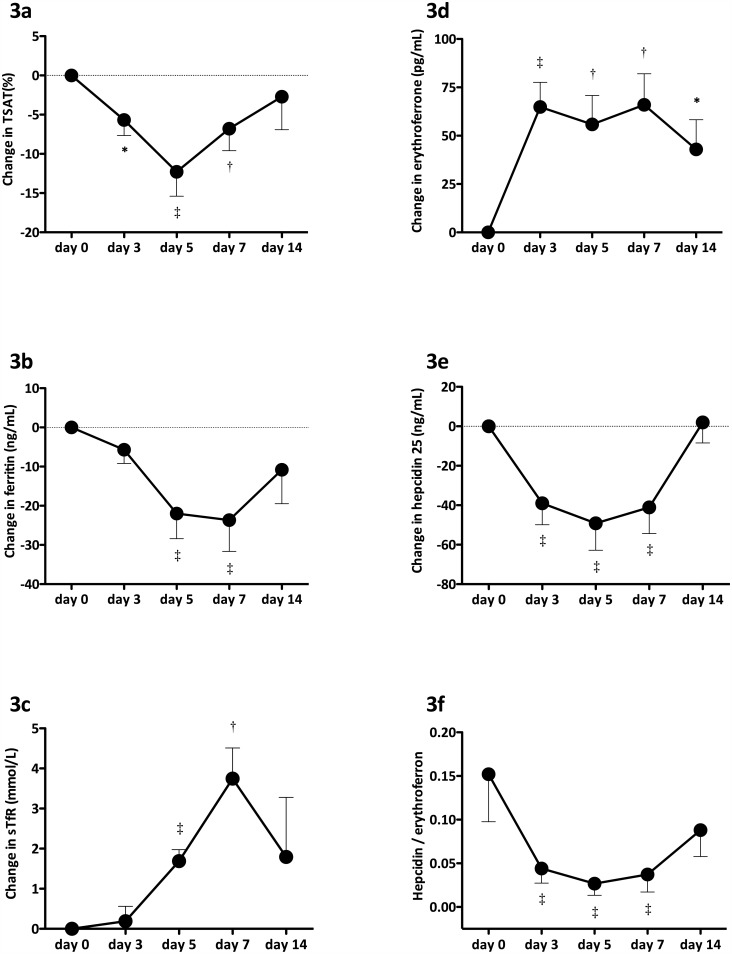
Changes in biomarkers of iron metabolism induced by continuous erythropoietin receptor activator. Biomarkers of iron metabolism; namely transferrin saturation (TSAT) (a), ferritin (b), soluble transferrin receptor (sTfR) (c), erythroferrone (d), hepcidin 25 (e) and ratio of hepcidin 25 to erythroferrone (f). Data are shown as means ± SEM. Baseline and time-point data were compared using Wilcoxon matched-pairs signed-rank test. *p < 0.05, ^†^p < 0.01, ^‡^p < 0.001 vs. baseline.

The TSAT was significantly reduced on day 5 after DA administration, and remained low until day 7 after CERA administration (Figs 2a and 3a). Ferritin levels did not differ between the DA and CERA groups and decreased on days 5 and 7 (Figs 2b and 3b). The sTfR levels tended to be elevated at day 7 in the DA group (Fig 2c), but values became significant between days 5 to 7 in the CERA group (Fig 3c). Levels of ERFE were increased and those of hepcidin 25 were decreased between days 3 and 5 compared with baseline in the DA group (Fig 2d and 2e). Ratios of hepcidin 25 to ERFE were decreased at day 3 (Fig 2f). Levels of ERFE were significantly increased between days 3 and 14 in the CERA group (Fig 3d). Hepcidin 25 levels were significantly lower than baseline in the CERA group at day 5 and increased to the baseline level at day 14 (Fig 3e). Ratios of hepcidin 25 to ERFE were significantly decreased at day 5 in the CERA group compared with baseline (Fig 3f). Baseline ERFE levels after ESA administration increased by 15%– 20%.

## Discussion

The present study examined associations between ERFE, a functional regulator of hepcidin 25 during erythropoiesis, with biomarkers of iron metabolism during erythropoiesis in patients under HD. Levels of ERFE and hepcidin 25 negatively correlated. The association of hepcidin 25 with ferritin was similar to that between the ratios of hepcidin 25 to ERFE with ferritin. Thus ERFE significantly associated with biomarkers of iron metabolism in patients under HD.

Several studies have confirmed that ESA affects iron metabolism [3–6]. We recently showed that the long-acting ESA, DA and CERA, decrease hepcidin 25 levels in patients under HD [6]. Here, levels of ERFE were obviously increased on day 3 compared with baseline after the intravenous administration of DA and CERA and those of hepcidin 25 changed on day 3 in parallel with the increase in ERFE. Changes in ferritin, sTfR, as well as reticulocyte count, were delayed compared with those in EFN and hepcidin 25. Kautz et al. confirmed that ESA stimulation alters levels of ERFE and hepcidin 25; ERFE is produced and released shortly after ESA injection, then hepcidin 25 levels decrease after nine hours of ESA stimulation [7]. Thus, ERFE might be a key regulator of iron mobilization from body stores during erythropoiesis in patients under HD who are treated with ESA.

High hepcidin 25 levels not only decrease the release of stored iron, but also influence erythropoiesis [12]. Thus, hepcidin 25 might be an important regulator of iron homeostasis under erythropoiesis in patients on dialysis [2]. On the other hand, several reports have indicated that hepcidin 25 is not a reliable biomarker for monitoring iron release and storage in patients under HD [13, 14]. Because ERFE plays roles in hepcidin 25 suppression and iron overload during erythropoiesis [7, 8] it might be useful for monitoring iron metabolism. We found a correlation between ferritin and ERFE, but it was not any closer than that between ferritin and hepcidin 25. Moreover, we did not examine associations between ERFE and iron responsiveness during erythropoiesis. Thus, the present study cannot address the value of ERFE monitoring for managing iron in patients who are being treated for anemia.

Several limitations are associated with this non-randomized observational investigation of a small patient cohort and the cross-sectional study at baseline. A small sample size and the nature of a non-randomized design might have influenced the statistical findings. In a broader context, skeletal muscle produces ERFE (termed myonectin) encoded by the FAM132B gene [15]. Thus, we considered ERFE values as total ERFE, being derived from both skeletal muscle and erythroblasts. The short study period of 14 days prevented analysis of the long-term influences of DA or CERA on ERFE. Thus, further studies should clarify the effects of long-acting ESA on iron metabolism including ERFE. Patients who had started ESA therapy within the past six months were not included and thus the findings regarding the association between ESA and iron metabolism were limited to patients on HD who were undergoing maintenance ESA therapy. The monthly CERA doses were double the biweekly doses administered to patients. Thus, the high CERA dose might have influenced the present findings. Iron-deficient patients received iron supplementation according to guidelines [10] before and during the study period, which might have affected baseline data regarding erythropoiesis and iron metabolism as well as other findings during the study period. Finally, higher levels of hepcidin 25 and of ferritin are independent predictors of CVD events and mortality in patients under HD [12, 16]. Therefore, large prospective cohort studies are required to evaluate associations between ERFE and CVD events as well as mortality in such patients.

In conclusion, ERFE might be associated with iron metabolism in patients with HD. Both DA and CERA could increase ERFE levels resulting in the regulation of hepcidin 25 and iron mobilization from body stores during erythropoiesis.

## Supporting Information

S1 Fig Changes in hemoglobin and reticulocyte count induced by darbepoetin-α. Data are shown as means ± SEM. Baseline and time-point data were compared using Wilcoxon matched-pairs signed-rank test. *p < 0.05 and ^†^p < 0.01 vs. baseline.
(TIFF)

S2 Fig Changes in hemoglobin and reticulocyte count induced by continuous erythropoietin receptor activator. Data are shown as means ± SEM. Baseline and time-point data were compared using Wilcoxon matched-pairs signed-rank test. *p < 0.05 and ^†^p < 0.01 vs. baseline.
(TIFF)
