# Supplementary figures and images for: Associations among Erythroferrone and Biomarkers of Erythropoiesis and Iron Metabolism, and Treatment with Long-Term Erythropoiesis-Stimulating Agents in Patients on Hemodialysis
Source: PLoS One. 2016 Mar 15;11(3):e0151601. doi: 10.1371/journal.pone.0151601 (PMC4792384; doi:10.1371/journal.pone.0151601)

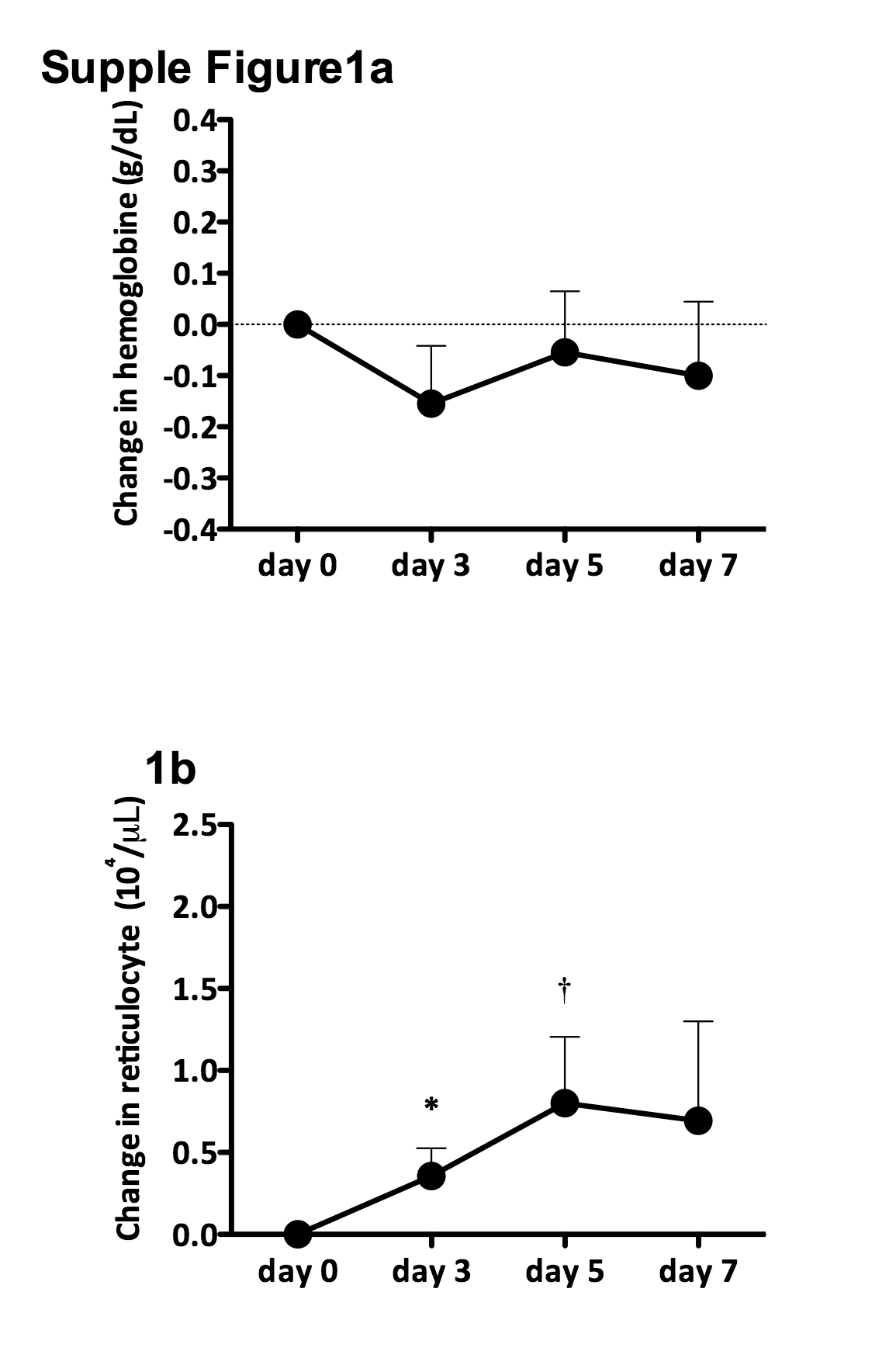

Supplement: S1 Fig — Data are shown as means ± SEM. Baseline and time-point data were compared using Wilcoxon matched-pairs signed-rank test. *p < 0.05 and †p < 0.01 vs. baseline. (TIFF) [file pone.0151601.s001.tiff]

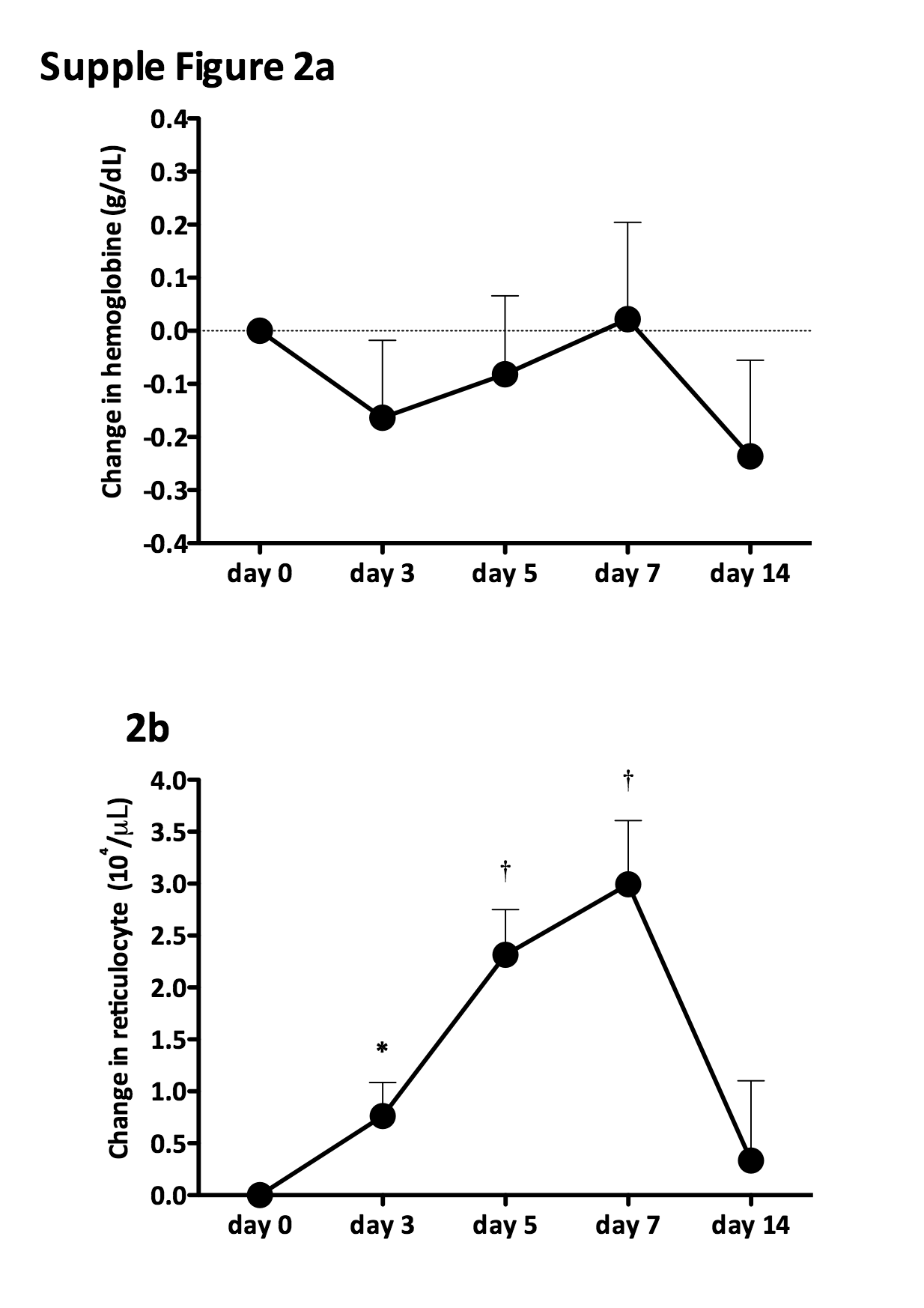

Supplement: S2 Fig — Data are shown as means ± SEM. Baseline and time-point data were compared using Wilcoxon matched-pairs signed-rank test. *p < 0.05 and †p < 0.01 vs. baseline. (TIFF) [file pone.0151601.s002.tiff]
